# Empowering Excellence: Creating Academic and Professional Parity in Graduate Medical Education

**DOI:** 10.1097/og9.0000000000000011

**Published:** 2024-05-30

**Authors:** Omar M. Young, Celeste A. Green, Jasmine D. Johnson

**Affiliations:** Division of Maternal-Fetal Medicine, Department of Obstetrics & Gynecology, UNC School of Medicine, Chapel Hill, North Carolina; the Division of Maternal-Fetal Medicine, Department of Obstetrics & Gynecology, Baylor College of Medicine, Houston, Texas; and the Division of Maternal-Fetal Medicine, Department of Obstetrics & Gynecology, Indiana University School of Medicine, Indianapolis, Indiana.

## Abstract

Reforms of graduate medical education in obstetrics and gynecology, along with honest conversations, are warranted to set up Black trainees for success.

We, Black physicians, are exhausted.

The road to graduate medical education and beyond for Black medical students is rather tortuous. Black medical students are less likely to know how to successfully navigate the residency match process and less likely to matriculate and complete a residency program.^1^ Segregation and the systematic dissolution of Black medical schools after the Flexner Report engendered today's dearth of Black physicians, who comprise only 5% of America's physician workforce.^2,3^ This scarcity limits generational support and role modeling for present-day medical students.^4–6^ When Black medical students do successfully matriculate to residency, they are often subjected to harsher, unwarranted scrutiny of their clinical performance and professionalism, leading to higher dismissal rates from training programs compared with White trainees.^7^

Being Black in medicine should not be painful and isolating. Yet, for so many of us, it is. American medical education was not designed to optimize our success. The foundation of systemic racism on which the American health care system was built not only harms our patients, but also those who are entrusted to “do no harm.” This cannot continue.

It seems like every news cycle features another attack on efforts to rectify long-standing inequities in medical training and practice, with claims that DEI (diversity, equity and inclusion) work promotes separation and distrust between racial and ethnic groups.^8,9^ These claims are proffered by individuals who have not felt the ill effects of being isolated, mistreated, unfairly disciplined, and undercompensated and who never have to worry about their achievements being called a fluke or a credit to their race.

We demand action from medical schools, academic medical centers, and residency programs to investigate how they can be agents of change—or how they are hindering it. Here is what that action could look like.

## SELF-ASSESSMENT

Kendi^10^ notes that, to be anti-racist, one must persistently be self-aware, self-critical, and in a constant state of self-examination. It may be that a program's existing culture reinforces environments that are ill-equipped to support Black trainees (at best) or that threaten their safety (at worst). Having open and honest conversations with patients, nurses, faculty, residents, fellows, and students enables everyone to have a voice and allows individuals to examine how their behavior augments or devalues a culture of inclusivity for all.

Many times, the duty of creating a structure for this self-assessment is placed on historically marginalized faculty. These faculty receive little or no departmental funding or protected time for their efforts. A full organizational restructuring—from vision to values—should be the goal. This will therefore require non-Black and nonminority faculty to buy in and play an active role in creating a new anti-racist culture. Black and other minority faculty have for too long been overburdened by these responsibilities.^11^

Employing outside facilitators may be the best way to ensure candor and objective conversation. Similar to strategies used to investigate health disparities, organizational climate surveys—when administered in a systematic and intentional way—can provide powerful insight into a department's existing cultural structure.

## MENTORSHIP AND SPONSORSHIP

Compared with their White counterparts, Black trainees have decreased exposure to medicine in their formative years and limited opportunities to see real-world applications of clinical practice. This contributes to a compromised professional-identity formation.^12^ The absence of role-modeling contributes to higher rates of burnout, isolation, and exclusion.^7^ For Black trainees to succeed, they need effective mentorship and sponsorship.

Often, Black trainees will seek an affirming professional relationship with a racially concordant mentor. Racial concordance may provide certain intangible benefits, because their mentor may more likely have shared cultural relatedness, which helps to build trust and understanding. However, racial discordance does not absolve non-Black faculty from the responsibility of mentorship to Black trainees. In fact, all faculty should be prepared to adequately assess and address the needs of Black trainees, to spare Black faculty and other faculty underrepresented in medicine from being overwhelmed by this academic burden. Additionally, with Black physicians comprising only 3.6% of all faculty, it may be impossible for Black trainees to find Black mentors at their home institutions.^12,13^ Those who shoulder the predominance of mentor–mentee relationships for underrepresented in medicine students should be rewarded for their efforts when it comes time for promotion and tenure. It is imperative that they receive support for professional development in preparing to be effective mentors. Moreover, national governing bodies for individual medical specialties and the Accreditation Council for Graduate Medical Education should consider the creation of national mentorship programs. Through connections at regional or national meetings, as well as through virtual platforms, Black trainees can bridge the mentorship gap and obtain what they need to succeed.

Black trainees can also assuredly benefit from sponsorship. Sponsorship comes from an attending or administrator vouching for someone based on merit and belief in their potential; it is pulling a chair up for that trainee to have a proverbial seat at the table.^11^ Having faculty create these opportunities for advancement can positively alter the course of their trainees' careers. Having a sponsor positions the Black trainee to be competitive with those who already have such access. Thus, both mentorship and sponsorship of Black trainees engenders academic equity and provides parity in medical education.

## CHANGING NORMS

Medicine is a very traditional and, at times, antiquated industry. What started as a brotherhood among White men now includes (though in inequitable representation) people who identify with all genders, races, ethnicities, abilities, and sexualities. We are still rooted in the “traditions” that had served as rites of passage and are ways to further segregate and differentiate. It is seen in the language used in evaluations peppered with racism to suggest inferiority in performance. It is felt in the painfully brushed off interactions and conflicts with nursing when the person writing orders that they have to follow for the first time has brown skin. It is in the fumbles of faculty when a patient requests care by certain “types” of physicians. Membership in medical fraternities, honor organizations, and selection of individuals for leadership roles too often relies on criteria rooted in systematic discrimination. Though deemed as adequate to those who are well-served by these traditional systems, data will speak to how a foundation of structural racism manifests in disparate outcomes from the Medical College Admissions Test to the residency match.^13^

We need to critically evaluate 1) how bias enters into letters of recommendation and rotation evaluations written for Black trainees, 2) how they are evaluated for promotion, and 3) how institutions can undertake concerted efforts to provide objective formative and summative assessment. Holistic review should be a tenet at all levels of medical training, not just when it comes to reviewing residency applications. The uniqueness about an applicant that is lauded as a much-needed change must not be used against them.

We hope that academic medical centers, medical schools, residency programs, and national leaders in medical education understand the gravity of this situation. For too long, Black trainees have been suffering in silence. It is time that suffering is given a voice. It is time that Black trainees are given the agency to realize the fullness of their potential.

We are tired of being exhausted.
